# Comparative analysis of the vertebral pneumatization in pterosaurs (Reptilia: Pterosauria) and extant birds (Avialae: Neornithes)

**DOI:** 10.1371/journal.pone.0224165

**Published:** 2019-10-25

**Authors:** Richard Buchmann, Leonardo dos Santos Avilla, Taissa Rodrigues

**Affiliations:** 1 Laboratório de Paleontologia, Departamento de Ciências Biológicas, Centro de Ciências Humanas e Naturais, Universidade Federal do Espírito Santo, Vitória, ES, Brazil; 2 Laboratório de Mastozoologia, Departamento de Zoologia, Universidade Federal do Estado do Rio de Janeiro, Rio de Janeiro, RJ, Brazil; 3 Programa de Pós-graduação em Ciências Biológicas (Biodiversidade Neotropical), Universidade Federal do Estado do Rio de Janeiro, Rio de Janeiro, RJ, Brazil; State Museum of Natural History, GERMANY

## Abstract

Birds and pterosaurs have pneumatic bones, a feature likely related to their flight capabilities but whose evolution and origin is still poorly understood. Pneumatic foramina are present on the external surface of the bone and are reliable indicators of post-cranial skeletal pneumatization present in Pterosauria, Eusauropoda, and Neotheropoda. Here, we carried out a qualitative analysis of the position, size and number of pneumatic foramina of the cervical and thoracic/dorsal vertebrae of pterosaurs and birds, as they have the potential to challenge hypotheses about the emergence and evolution of the respiratory trait in these groups. We also discussed differences between pneumatic and vascular foramina for identification purposes. Besides phylogenetic representativeness, the pterosaur taxonomic sampling considered the preservation of specimens and, for birds, their life habit, as this relates to the level of pneumatization. Pneumatic foramina on the lateral faces of the centrum of the mid-cervical vertebrae of pterosaurs and birds differ in position and size, and those adjacent to the neural canal additionally differ in number. The avian posterior cervical vertebrae show a higher number of pneumatic foramina in comparison to their mid-cervicals, while the opposite is true for pterosaurs, suggesting differences in the cervical air sac of these clades. Pneumatic foramina were found at the base of the transverse processes of the notarial vertebrae of birds, while they were absent from some of the pterosaurs analyzed here, revealing the presence of a pneumatic hiatus in the vertebral column that might be explained due to the distance of this structure to the cervical air sac. These findings indicate that, although the overall skeletal pneumatization of pterosaurs and birds present deep homologies, some pneumatic features occurred convergently because variation in the number of pneumatic foramina along the vertebral column is related to the position of the air sacs in pterosaurs and birds and/or the habit of each species. There is an evident reduction of the pneumatic foramina in birds that have aquatic foraging and an increase in the ones which perform static soaring. Although we did not find any external anatomical difference between pneumatic and vascular foramina, we observed that vascular foramina occur at specific sites and thus identification on the basis of location is reliable.

## Introduction

Nowadays birds are the only extant tetrapods with post-cranial skeletal pneumatization, a condition correlated to the presence of the air sacs that are part of their respiratory system [1]. The intake of air to the bones happens through the pneumatic foramina, which form air diverticula in the bone intratrabecular cavity [2]. Although pneumatic foramina and fossae are reliable cortical indicators of skeletal pneumatization [3], fossae may be associated with other tissues, such as muscles and adipose tissue, and foramina can be pneumatic, vascular, or nervous [4]. Currently, it is considered that for a foramen to be unequivocally identified as pneumatic it must be connected to the internal intertrabecular cavities [4]. As indicators of skeletal pneumatization, these foramina allow inferences on the presence of pneumatic bones in extinct animals [4,5].

Given the presence of pneumatization in non-avian dinosaurs and pterosaurs, two different hypotheses about the origin of bone pneumatization are discussed: it might have arisen once in a non-ornithodiran archosaur ancestor, or it might have appeared two times independently in the evolutionary history of Ornithodira [6–8].

Here, we compare the distribution, shape and size of pneumatic foramina present in the vertebral skeleton of pterosaurs and extant birds in order to test the before-mentioned hypotheses, taking into account the known life habit of each species, because this is a factor that influences the presence of pneumatization [3]. We also checked for diagnostic characteristics of pneumatic foramina, which have the potential to make this structure more easily identifiable.

## Material and methods

### Analyzed specimens

The analyzed pterosaurs come from the Romualdo Formation, in the Araripe Basin in northeastern Brazil. The three-dimensional preservation of the specimens recovered in this lithostratigraphic unit allows a more precise visualization of the cortical foramina that are probably connected to internal pneumatic cavities. The vertebrae belong to members of the families Anhangueridae (*Anhanguera piscator* replica MN 5023-V; *Tropeognathus* cf. *mesembrinus*, MN 6594-V) and Tapejaridae, including Tapejarinae (AMNH 24445) and Thalassodrominae (AMNH 22568, MN 4728-V, MN 6504-V, and MN 6566-V), as well as two undetermined Tapejaridae (MN 6588-V and MN 6511-V) [9–13]. One last specimen, not yet described in detail in the literature (MN 6508-V), could be identified only as Pterosauria sp.

The studied vertebrae of Neornithes comprised living species of the taxa Rheiformes (*Rhea americana*, MNA 766), Tinamiformes (*Tinamus solitarius*, AZ 136, AZ 138, and AZ 139), Anseriformes (*Cairina moschata*, AZ 763), Galliformes (*Pipile jacutinga*, AZ 767 and MNA 7664; *Penelope superciliaris*, MNA 2058), Procellariiformes (*Procellaria aequinoctiallis*, MNA 8553; *Diomedea chlororhynchus*, MNA 1793; *Pterodroma sp*., AZ 1196), Pelecaniformes (*Egretta caerulea*, AZ 1073; *Ardea alba*, MN 51321; *Egretta thula*, MNA 6993), Suliformes (*Phalacrocorax brasilianus*, MNA 2051; *Fregata magnificens*, MNA 1977; *Sula leucogaster*, MNA 7665), Cathartiformes (*Cathartes aura*, AZ 578 and AZ 580), Strigiformes (*Athene cunicularia*, AZ 496 and AZ 1384; *Megaschops choliba*, AZ 1545; *Glaucidium brasilianus*, AZ 323; *Asio clamator*, AZ 1190; *Tyto furcata*, AZ 1543), Falconiformes (*Milvago chimachima*, MNA 4305 and 5086; *Caracara plancus*, AZ 1297, MNA 4902, and MNA 4589; *Falco sparverius*, AZ 1547, AZ 1185, AZ 1548, AZ 1096 and AZ 1092), and Psittaciformes (*Ara macao*, MNA 001; *Ara ararauna*, AZUSP 040; *Ara chloropterus*, AZ 762; *Amazona amazonica*, AZ 066). Rheiformes and Tinamiformes were selected because they represent basal clades of Neornithes, and Galliformes and Anseriformes represent basal clades of Neognathae [14]. The other clades were analyzed due to their different foraging activities and flight habits. Procellariformes and Suliformes have a more gliding flight habit and forage in deeper waters. Pelecaniformes have an active flight and forage in shallow waters. Falconiformes have both flapping and gliding flight and forage terrestrially. Strigiformes and Psittaciformes have an active flight habit and show terrestrial foraging, and Cathartiformes have a gliding flight and a scavenging feeding habit [15–18].

### Descriptive terms

Only cervical (excluding the atlas and axis) and trunk (in pterosaurs, normally called dorsal) vertebrae were considered, because sacral and caudal vertebrae are normally not pneumatized in pterosaurs. Pterosaur vertebrae were termed following Bennett [19]: ‘mid-cervical vertebrae’, referring to cervicals 3–7; ‘posterior cervical vertebrae’, to cervicals 8 and 9 (i.e., the last two vertebrae of the cervical series); ‘notarium’, when such structure is present, normally from the 9^th^ cervical to the 4^th^ dorsal vertebrae; and ‘free dorsal vertebrae’ are the dorsals that do not take part on the notarium. Bird vertebrae were classified after Baumel and Witmer [20] and termed to propose their correspondence to the pterosaur homologues: ‘mid-cervical vertebrae’ refer to the second neck segment, from the 5^th^ or 6^th^ vertebrae to the 9^th^ or 11^th^, depending on the species [21]. ‘Posterior cervical vertebrae’ refer to the third cervical segment, from the 10^th^ or 11^th^ to the 13^th^ or 15^th^ vertebrae [21]. The ‘notarium’, when present, is composed by 2 to 6 fused thoracic vertebrae; and ‘free thoracic vertebrae’ were termed for the thoracic vertebrae that do not participate on the notarium [definition after 20].

**Fig 1:**
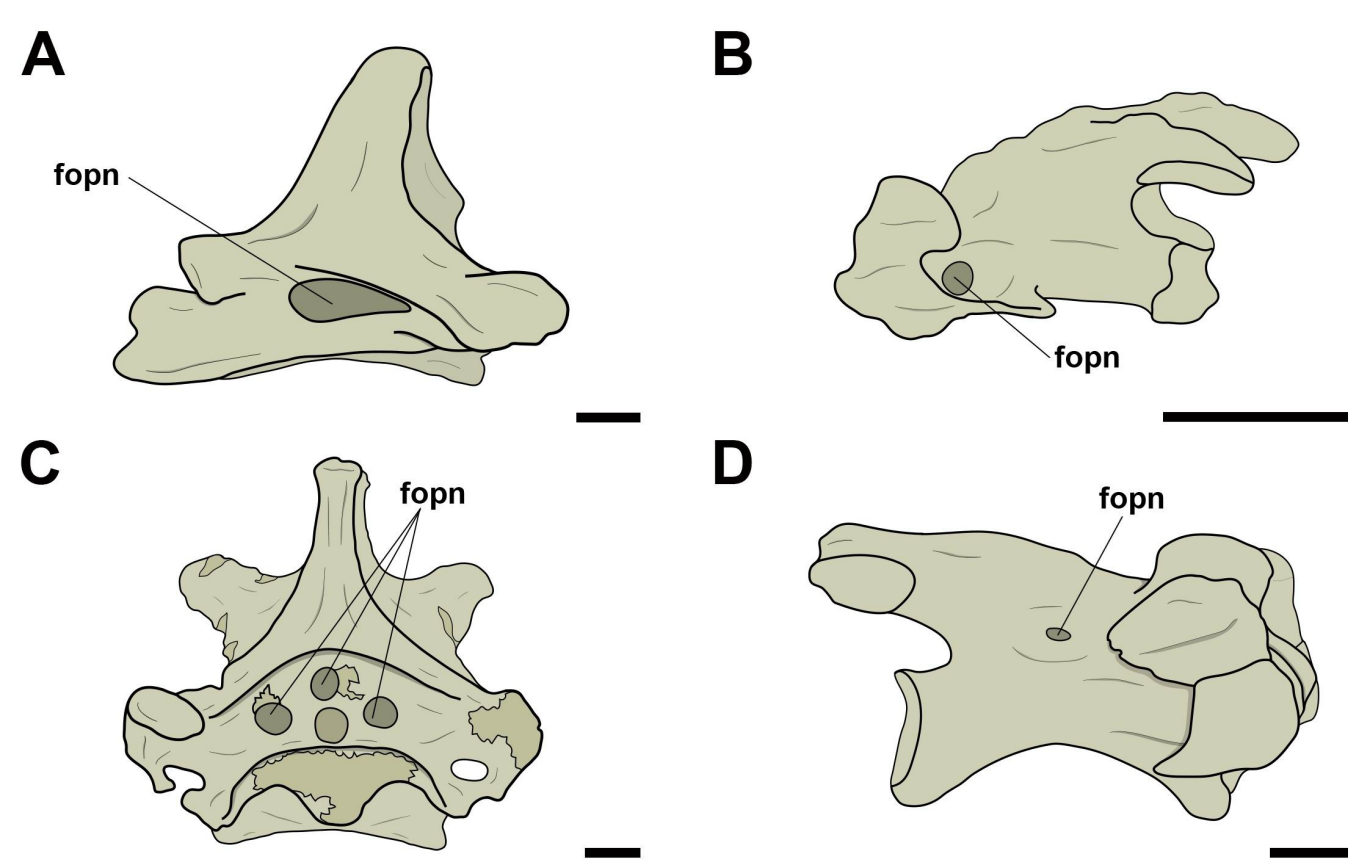
Pneumatic foramina observed in cervical vertebrae of pterosaurs and extant birds. A, Lateral pneumatic foramen in right lateral view; B, craniolateral pneumatic foramen in left lateral view; C, pneumatic foramina adjacent to the neural canal in cranial view; D, pneumatic foramina on the lateral side of the neural arch in right lateral view. Abbreviations: fopn, pneumatic foramen. Scale: 10 mm.

Concerning the pneumatic foramina in cervical vertebrae, we follow the nomenclature proposed by Vila Nova et al. [12], in which the ones present on the lateral cortical surface of the centrum are referred to as ‘lateral pneumatic foramina’ (Fig 1A). Thus, foramina observed in a craniolateral position in relation to those were named ‘craniolateral pneumatic foramina’ (Fig 1B). Still based on the nomenclature proposed by Vila Nova et al. [12], all pneumatic foramina present laterally and dorsally to the neural canal, both in pterosaurs and birds, were denominated as ‘pneumatic foramina adjacent to the neural canal’, regardless of their number (Fig 1C). ‘Pneumatic foramina seen on the lateral of the neural arch’ were termed as such (Fig 1D).

**Fig 2:**
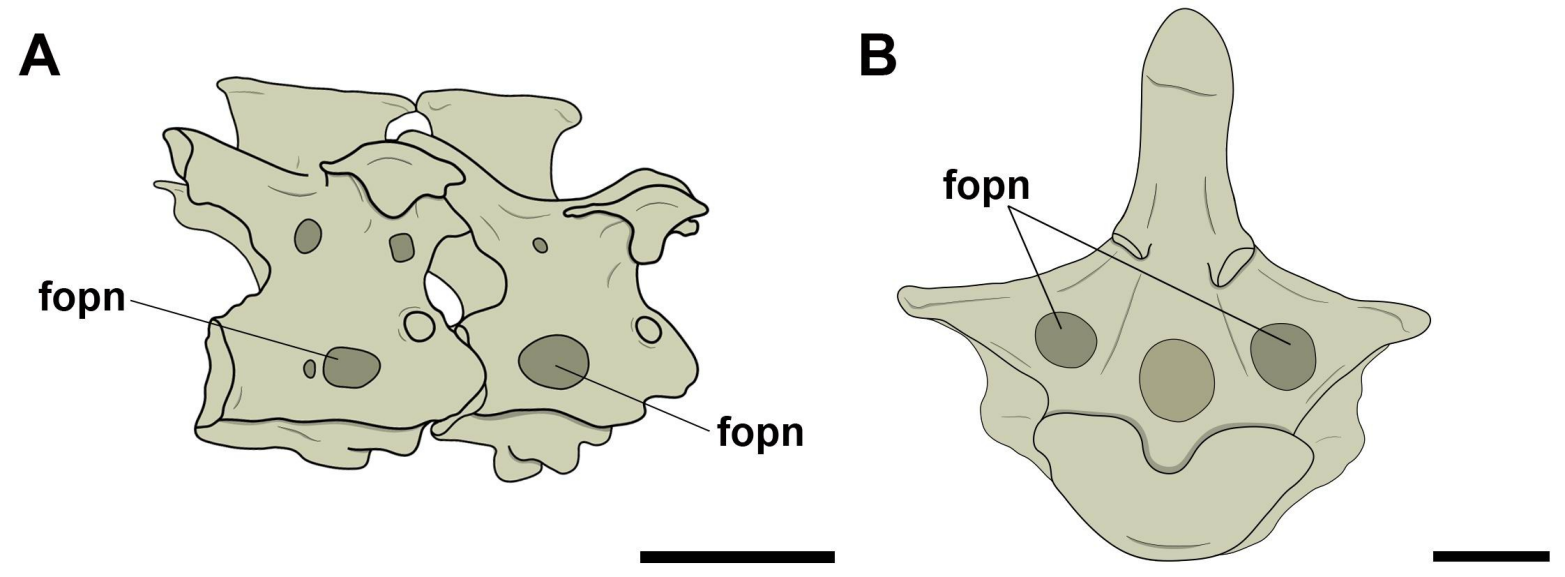
Pneumatic foramina observed in dorsal/thoracic vertebrae of pterosaurs and extant birds. A, Pneumatic foramen on the lateral face of the centrum in right lateral view; B, pneumatic foramen at the base of the transverse process in posterior view. Abbreviations: fopn, pneumatic foramen. Scale: 10 mm.

On the free dorsal/thoracic vertebrae and the notarium, we consider the term ‘lateral pneumatic foramina’ for all those found on the lateral surface of the centrum. We frequently observed structures, in cranial and/or caudal view, which are referred to as ‘pneumatic foramina present at the base of transverse processes’, because they, unlike the adjacent foramina seen in cervical vertebrae, lie in a more lateral position, considerably closer to the transverse processes, even though they are commonly close to the neural canal due to their size (Fig 2). We have not observed pneumatic foramina on the ventral surfaces of the presacral vertebrae in either pterosaurs or birds.

We follow the nomenclature established by Wilson et al. [22] in regard to the pneumatic fossae present on the neural arch and on the centrum of the vertebrae of Sauropodomorpha, which is also suitable for other ornithodirans.

## Results

### Pterosaurs

Table 1 summarizes the presence or absence of the main pneumatic foramina along the vertebral segments of the analyzed pterosaurs.

**Table 1:** Occurrence of pneumatic foramina on the vertebrae of pterosaurs. + indicates the presence of a foramen;–indicates the absence of a foramen; ? indicates an unknown condition. Abbreviations: fopn, pneumatic foramen.

Species/fopn	Mid-cervical; centrum; lateral	Mid-cervical; neural canal; adjacent	Posterior cervical; centrum; lateral	Posterior cervical; neural spine; base	Free dorsal; transverse process; base
***Anhanguera piscator* (MN 5023-V)**	+	+	+	–	+
***Tropeognathus* cf. *T*. *mesembrinus* (MN 6594-V)**	+	–	+	–	?
**Tapejarinae sp. (AMNH 24445)**	+	+	?	?	?
**Thalassodrominae sp. (AMNH 22568)**	+	+	+	+	?
**Thalassodrominae sp. (MN 4728-V)**	+	+	?	?	?
**Thalassodrominae sp. (MN 6566-V)**	+	+	+	+	?
**Thalassodrominae sp. (MN 6504-V)**	+	?	?	?	?
**Tapejaridae sp. (MN 6511-V)**	+	+	?	?	?
**Tapejaridae sp. (MN 6588-V)**	?	?	?	?	–
**Pterosauria sp. (MN 6508-V)**	?	?	?	?	+

Most foramina observed in this clade had a connection to the intertrabecular cavities and were thus identified as pneumatic. However, in some of them it was not possible to observe the connection between the cortex and the intertrabecular cavities, due to preservation issues or the presence of sediment. Because they were located in the same places as other pneumatic foramina, they were considered as such.

#### Mid-cervical vertebrae

**Fig 3:**
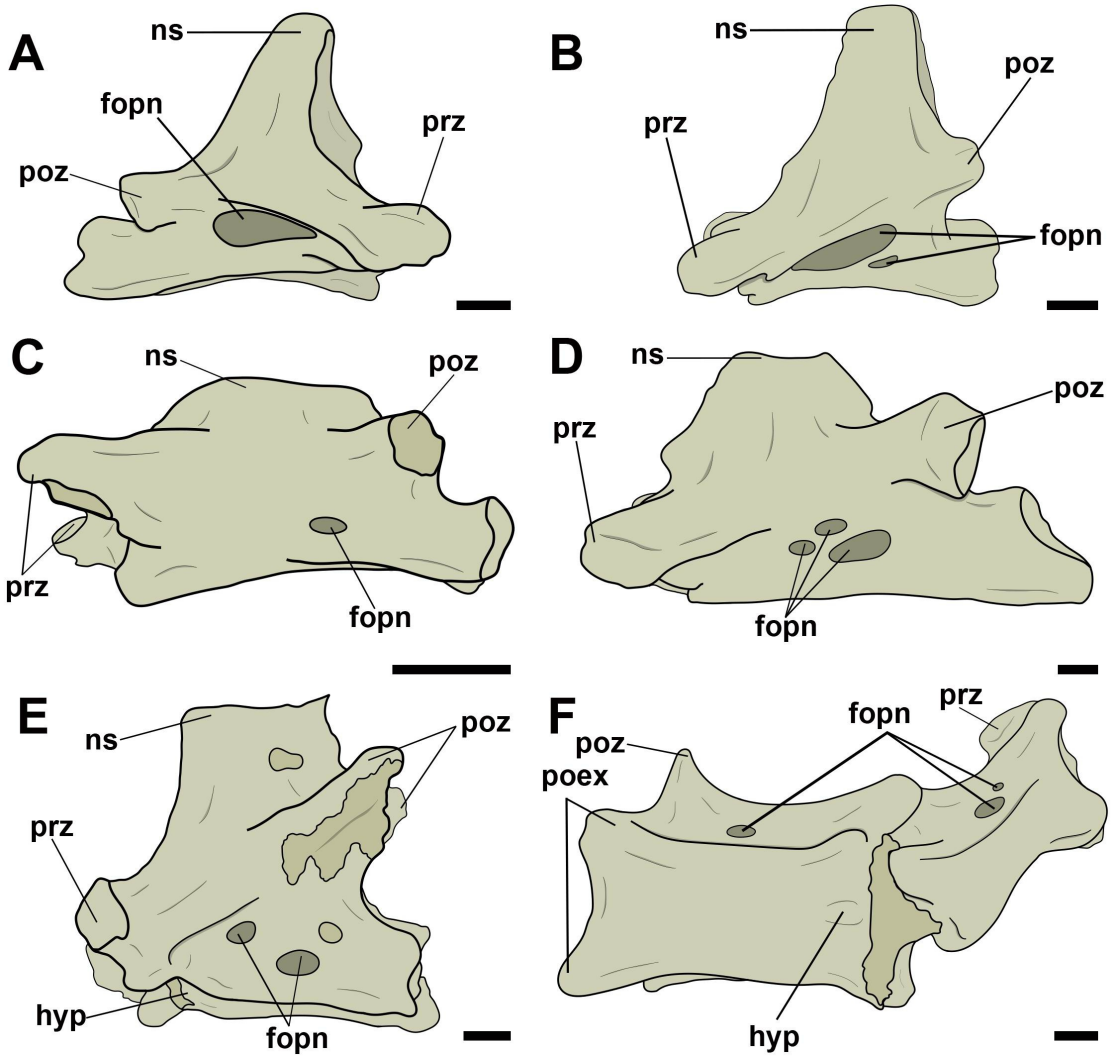
Lateral pneumatic foramina located on the centra of mid-cervical vertebrae in pterosaurs. A and B, *Anhanguera piscator* (MN 5023-V) in right and left lateral view, respectively; C, Tapejarinae sp. (AMNH 24445) in left lateral view; D, Thalassodrominae sp. (AMNH 22568) in left lateral view; E, Thalassodrominae sp. (MN 6566-V) in left lateral view; F, Thalassodrominae sp. (MN 6504-V) in ventro-lateral view. Abbreviations: fopn, pneumatic foramen; hyp, hypapophysis; poex, postexapophysis; ns, neural spine; poz, postzygapophysis; prz, prezygapophysis. Scale: 10 mm.

The mid-cervical vertebrae of all observed specimens had at least one pneumatic foramen on the right and left sides of the lateral surface of the vertebral centrum. Independent of the different sizes of the vertebrae, which varied between taxa, these foramina had a long oval outline. Although there is usually only one lateral pneumatic foramen, some vertebrae had two, which were located exclusively on the cervical vertebrae V, VI and VII, as is observed in the anhanguerids MN 5023-V and MN 6594-V and in the tapejarids MN 6566-V, MN 6504-V, and MN 6511-V (Fig 3). In a single case, we observed three lateral foramina, on the left side of cervical vertebra V of AMNH 22568, attributed to Thalassodrominae. The other side, however, showed only two foramina, so this configuration is likely an individual variation.

**Fig 4:**
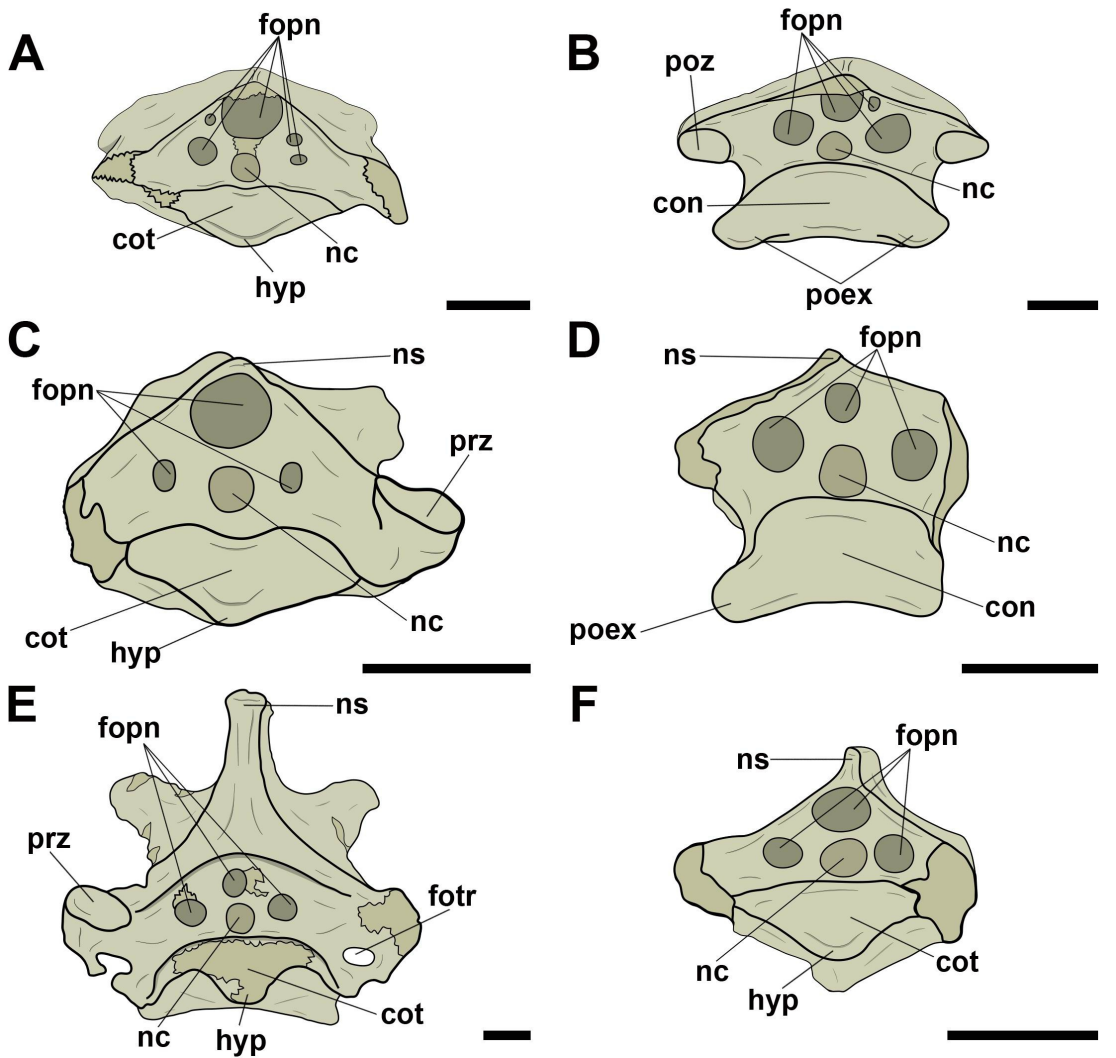
Pneumatic foramina located adjacent to the neural canal of mid-cervical vertebrae in pterosaurs. A, and B, Tapejaridae sp. (MN 6511-V) in cranial and caudal view, respectively; C and D, Thalassodrominae sp. (MN 4728-V) in cranial and caudal view, respectively; E, Thalassodrominae sp. (MN 6566-V) in cranial view; F, Tapejarinae sp. (AMNH 24445) in cranial view. Abbreviations: con, condyle; cot, cotyle; fopn, pneumatic foramen; fotr, *foramen transversarium*; hyp, hypapophysis; nc, neural canal; ns, neural spine; poex, postexapophysis; poz, postzygapophysis; prz, prezygapophysis. Scale: 10 mm.

Pneumatic foramina adjacent to the neural canal were observed in cranial and/or caudal view in all vertebrae whose preservation allowed viewing (Fig 4). The foramina of specimen MN 5023-V, replica of the holotype of *Anhanguera piscator*, were found only laterally to the neural canal along the cervical series. Additionally, in all analyzed tapejarids, the pair of pneumatic foramina laterally adjacent to the neural canal was accompanied by a foramen dorsal to the neural canal. One case of asymmetry in the distribution of the foramina was observed: the specimen MN 6511-V (Tapejaridae sp.) showed two foramina on the right side of the neural canal, each one of different sizes, while there was only one foramen on the left of the canal. We interpret this as an individual variation.

#### Posterior cervical vertebrae

On the posterior cervical vertebrae, we observed that the pneumatic foramina on the lateral faces of the centra were reduced, and even absent in some specimens. It is important to consider that these vertebrae also have shortened vertebral centra in relation to the mid-cervicals, being morphologically very similar to the first dorsal vertebrae [19]. Posterior cervical vertebrae also showed pneumatic foramina adjacent to the neural canal in cranial and/or caudal view in all specimens we could analyze. However, the foramen positioned dorsally to the neural canal, present in the mid-cervical vertebrae of the Tapejaridae, was absent in the posterior cervicals.

**Fig 5:**
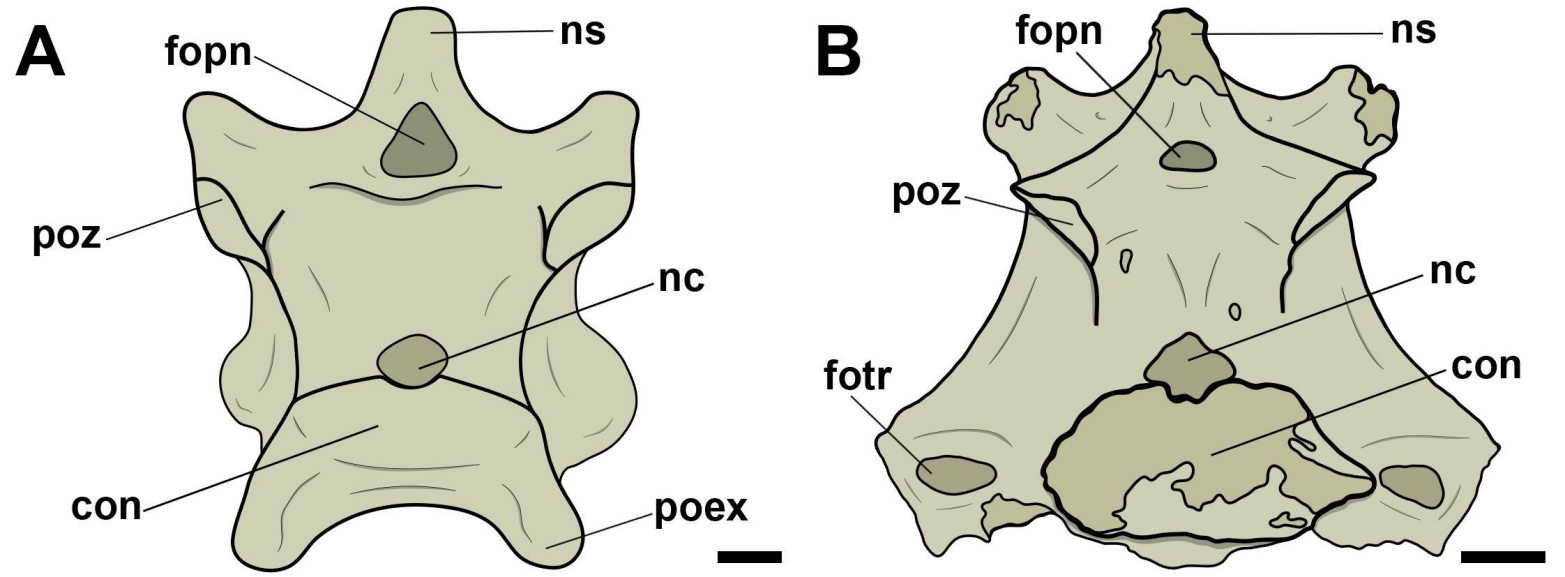
Pneumatic foramen located at the base of the neural spine in posterior cervical vertebrae in pterosaurs. A, Thalassodrominae sp. (AMNH 22568) in caudal view; B, Thalassodrominae sp. (MN 6566-V) in caudal view. Abbreviations: con, condyle; fopn, pneumatic foramen; fotr, *foramen transversarium*; nc, neural canal; ns, neural spine; poex, postexapophysis; poz, postzygapophysis. Scale: 10 mm.

One unusually placed pneumatic foramen was observed in the posterior cervical vertebrae of the thalassodromine MN 6566-V described by Buchmann et al. [13], and is also seen in AMNH 22568. It is located at the base of the neural spine, between the postzygapophyses in dorsocaudal view (Fig 5).

We observed adjacent pneumatic foramina located laterally to the neural canal in anterior view, and on the bases of the transverse processes in posterior view, in the posterior cervical vertebrae of *Anhanguera piscator* (MN 5023-V). These foramina had a circular outline and were always smaller than the neural canal. No external evidence of pneumatization was found on the centra of the posterior cervical vertebrae of the analyzed species.

#### Notarium

In dsungaripteroid pterosaurs, the notarium forms in osteologically mature specimens [23]. In this analysis, only the species *Tropeognathus* cf. *mesembrinus* (MN 6594-V) and a specimen of Tapejaridae (6588-V) have preserved notaria, but only in the second one pneumatic foramina at the base of transverse processes were found.

#### Free dorsal vertebrae

**Fig 6:**
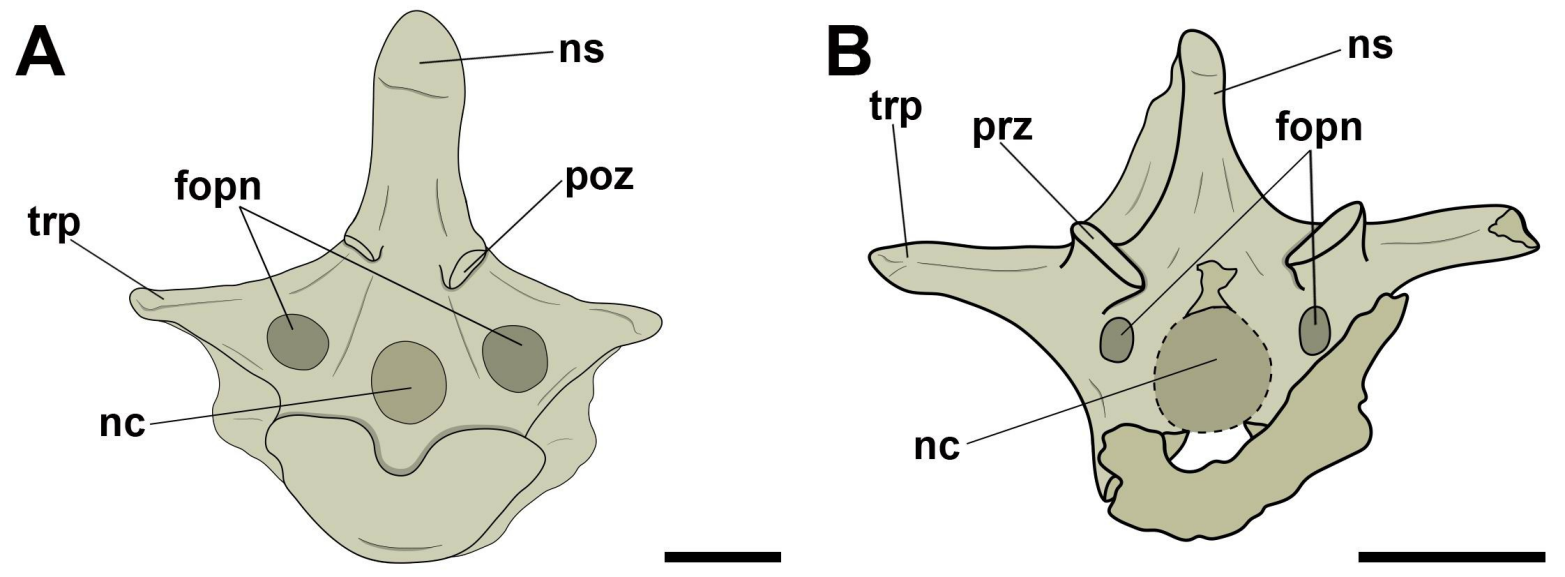
Pneumatic foramina located at the base of transverse processes of dorsal vertebrae in pterosaurs. A, *Anhanguera piscator* (MN 5023-V) in caudal view; B, Pterosauria sp. (MN 6508-V) in cranial view. Abbreviations: fopn, pneumatic foramen; nc, neural canal; ns, neural spine; poz, postzygapophysis; prz, prezygapophysis; trp, transverse process. Scale: 10 mm.

The third vertebra of the dorsal sequence of MN 5023-V (*Anhanguera piscator*) and one unidentified dorsal vertebra of the specimen MN 6508-V (Pterosauria sp.) showed pneumatic foramina located at the base of the transverse processes (Fig 6), similar to those seen in the posterior cervical vertebrae of MN 5023-V. No pneumatic foramina were seen on the centra of the free dorsal vertebrae of the analyzed specimens.

### Birds

Table 2 summarizes the presence or absence of the main pneumatic foramina along the vertebral segments of the analyzed birds. Among the analyzed material, only *Phalacrocorax brasilianus* (MNA 2051) had a drastic reduction in their number: it only showed one small oval pneumatic foramen at the base of the transverse processes on the right and left sides of the thoracic vertebrae. We also note that its vertebrae show extremely laterally compressed centra.

**Table 2:** Occurrence of pneumatic foramina on the vertebrae of birds. + indicates the presence of a foramen;–indicates the absence of a foramen. Individual variations are omitted for clarity. Abbreviations: fopn, pneumatic foramen.

Taxa/fopn	Mid-cervical; centrum; craniolaterally	Mid-cervical; neural canal; adjacent	Posterior cervical; neural arch; lateral	Posterior cervical; centrum; lateral	Free dorsal; transverse process; base	Free dorsal; centrum; lateral
***Rhea americana* (Rheiformes)**	+	–	+	–	+	–
***Tinamus solitarius* (Tinamiformes)**	+	+	+	–	+	–
***Cairina moschata* (Anseriformes)**	+	+	+	+	+	–
***Pipile jacutinga* (Galliformes)**	+	–	–	+	+	–
***Penelope superciliaris* (Galliformes)**	–	+	–	–	+	–
***Procellaria aequinoctiallis* (Procellariiformes)**	+	–	+	+	+	+
***Diomedea chlororhynchus* (Procellariiformes)**	+	+	+	+	+	+
***Pterodroma* sp. (Procellariiformes)**	+	+	+	+	+	–
***Egretta caerulea* (Pelecaniformes)**	+	–	–	–	+	–
***Ardea alba* (Pelecaniformes)**	–	–	–	–	+	–
***Egretta thula* (Pelecaniformes)**	+	–	–	–	+	–
***Phalacrocorax brasilianus* (Suliformes)**	–	–	–	–	–	–
***Fregata magnificens* (Suliformes)**	+	+	+	+	+	–
***Sula leucogaster* (Suliformes)**	+	+	+	+	+	+
***Cathartes aura* (Cathartiformes)**	+	–	+	+	+	–
***Athene cunicularia* (Strigiformes)**	+	+	–	–	+	–
***Megaschops choliba* (Strigiformes)**	+	–	–	–	+	–
***Glaucidium brasilianus* (Strigiformes)**	+	–	+	+	+	–
***Asio clamator* (Strigiformes)**	+	+	–	+	+	–
***Tyto furcata* (Strigiformes)**	+	+	+	+	+	–
***Milvago chimachima* (Falconiformes)**	+	+	+	+	+	–
***Caracara plancus* (Falconiformes)**	+	+	+	+	+	–
***Falco sparverius* (Falconiformes)**	+	+	+	+	+	–
***Ara macao* (Psittaciformes)**	+	+	–	+	+	–
***Ara ararauna* (Psittaciformes)**	+	+	+	+	+	+
***Ara chloropterus* (Psittaciformes)**	+	+	–	+	+	–
***Amazona amazonica* (Psittaciformes)**	+	–	–	+	+	–

Besides pneumatic foramina, most birds also possess foramina that are strictly vascular. They are frequently smaller and more oval than pneumatic ones, although size is not diagnostic. Vascular foramina are usually located laterally to the *foramen transversarium* in cervical vertebrae, as in *Rhea americana* and *Ara ararauna*, and associated with the ventral base of the transverse processes in thoracic vertebrae, as in *Ardea alba* and *Ara chloropterus*.

#### Mid-cervical vertebrae

**Fig 7:**
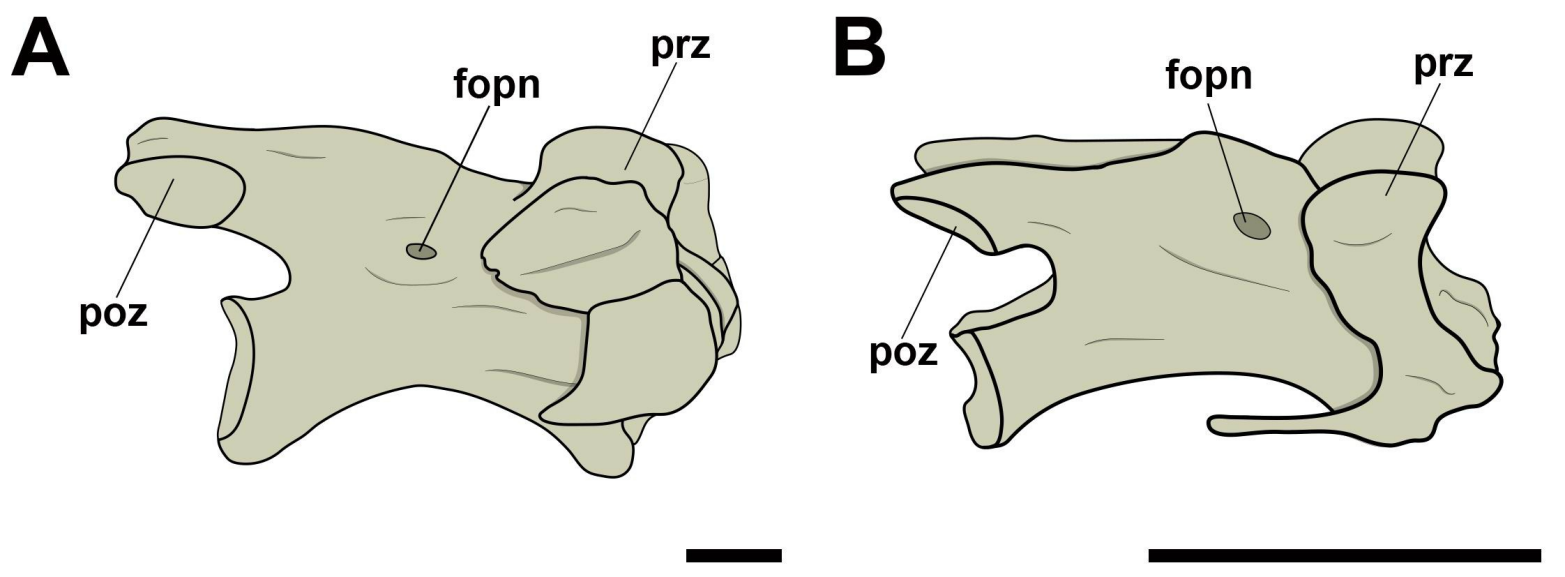
Pneumatic foramina located on the lateral surface of the mid-cervical vertebrae in paleognath birds. A, *Rhea americana* (MNA 766) in right lateral view; B, *Tinamus solitarius* (AZ 139) in right lateral view. Abbreviations: fopn, pneumatic foramen; poz, postzygapophysis; prz, prezygapophysis. Scale: 10 mm.

The analyzed paleognaths presented a pneumatic foramen in an unusual position at the neural arch compared to the neognaths, located on the lateral surface of the cranial portion of the neural arch (*Rhea americana*, MNA 766, and *Tinamus solitarius*, AZ 138 and AZ 139), and has a long and craniocaudally oval outline (Fig 7).

**Fig 8:**
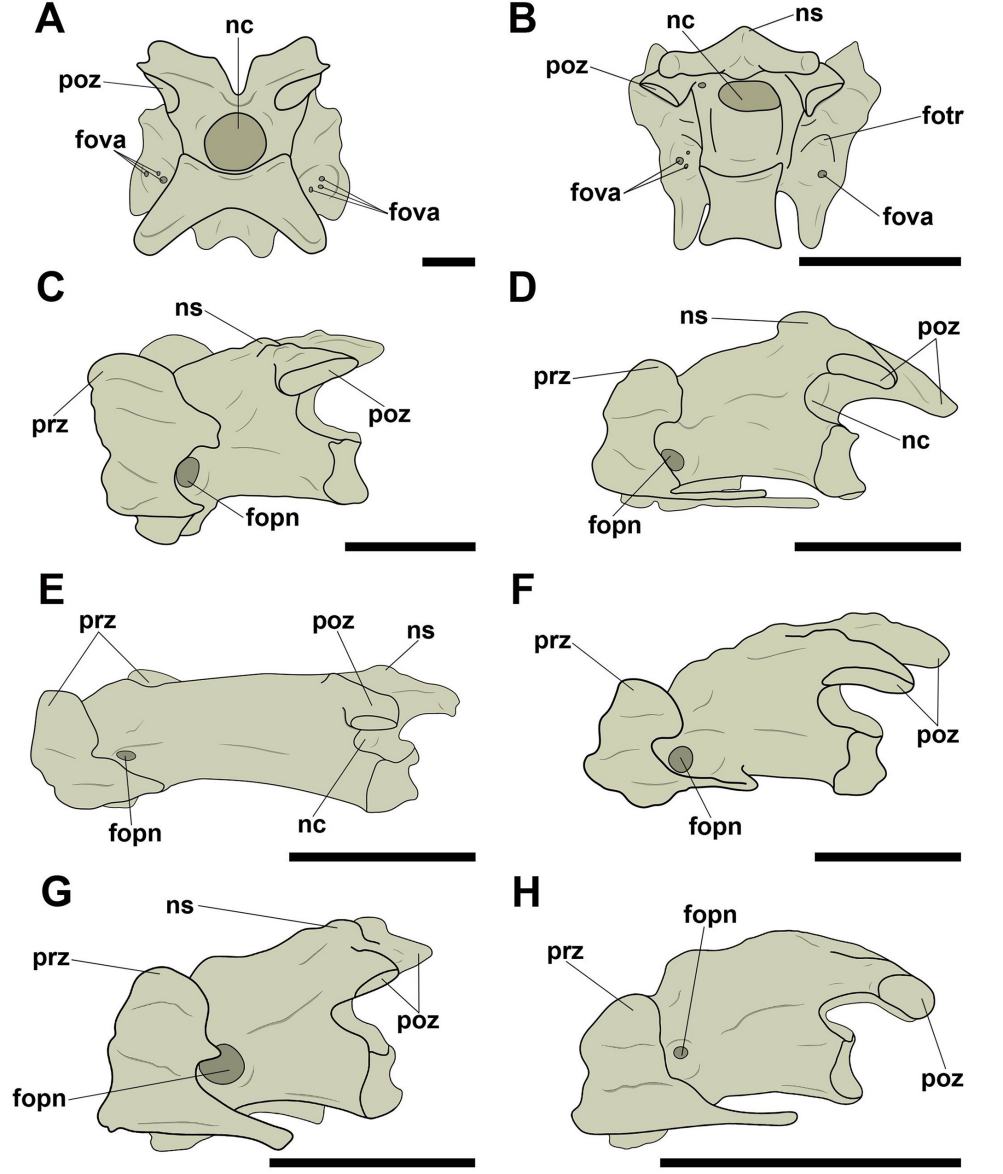
Pneumatic foramina located craniolaterally on the centra of mid-cervical vertebrae in birds. A, *Rhea americana* (MNA 766) in caudal view; B, *Ara ararauna* (AZUSP 040) in caudal view; C, *Diomedea chlororhynchus* (MNA 1793) in left lateral view; D, *Procellaria aequinoctiallis* (MNA 8553) in left lateral view; E, *Egretta thula* (MNA 6993) in left lateral view; F, *Fregata magnificens* (MNA 1977) in left lateral view; G, *Cathartes aura* (AZ 578) in left lateral view; H, *Asio clamator* (AZ 1190) in left lateral view. Abbreviations: fopn, pneumatic foramen; fotr, *foramen transversarium*; fova, vascular foramen; nc, neural canal; ns, neural spine; poz, postzygapophysis; prz, prezygapophysis. Scale: 10 mm.

All analyzed birds have at least one circular craniolateral pneumatic foramen on the right and left lateral faces of the mid-cervical vertebrae, close to the *foramen transversarium* (Fig 8). The absence of this pneumatic foramen is rare and observed only in a few species, such as *Penelope superciliaris* (MNA 2058) and *Ardea alba* (MN 51321), although this foramen has been observed in other Galliformes (*Pipile jacutinga*, MNA 7664) and Pelecaniformes (*Egretta thula*, MNA 6993, and *Egretta caerulea*, AZ 1073).

**Fig 9:**
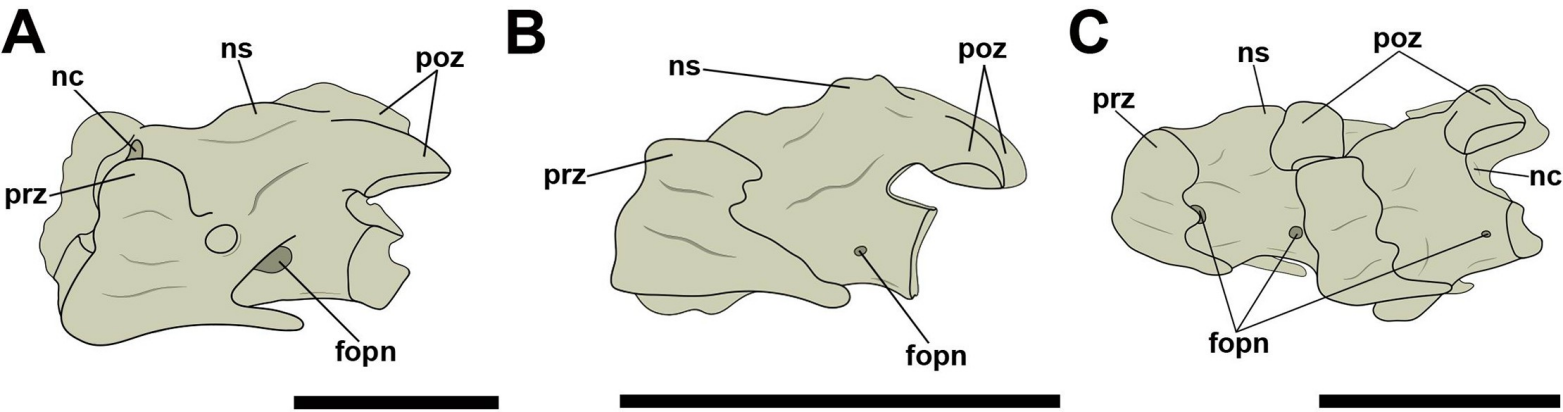
Pneumatic foramen located laterally on the centra of mid-cervical vertebrae in birds. A, *Cairina moschata* (AZ 763) in left lateral view; B, *Milvago chimachima* (MNA 5086) in left lateral view; C, *Amazona amazonica* (AZ 066) in left lateral view. Abbreviations: fopn, pneumatic foramen; nc, neural canal; ns, neural spine; poz, postzygapophysis; prz, prezygapophysis. Scale: 10 mm.

Some species belonging to Neognathae also present an additional pneumatic foramen on the right and left lateral faces of the vertebral centrum in a position more caudal than the craniolateral pneumatic foramen mentioned in the previous paragraph. When these pneumatic foramina were observed, the craniolateral ones were also present. Like the craniolateral foramina, they have a circular outline. These foramina are similar in shape and position across different taxa and were observed only in some analyzed species, among them *Cairina moschata* (AZ 763), *Milvago chimachima* (MNA 4305 and MNA 5086), and *Amazona amazonica* (AZ 066) (Fig 9).

**Fig 10:**
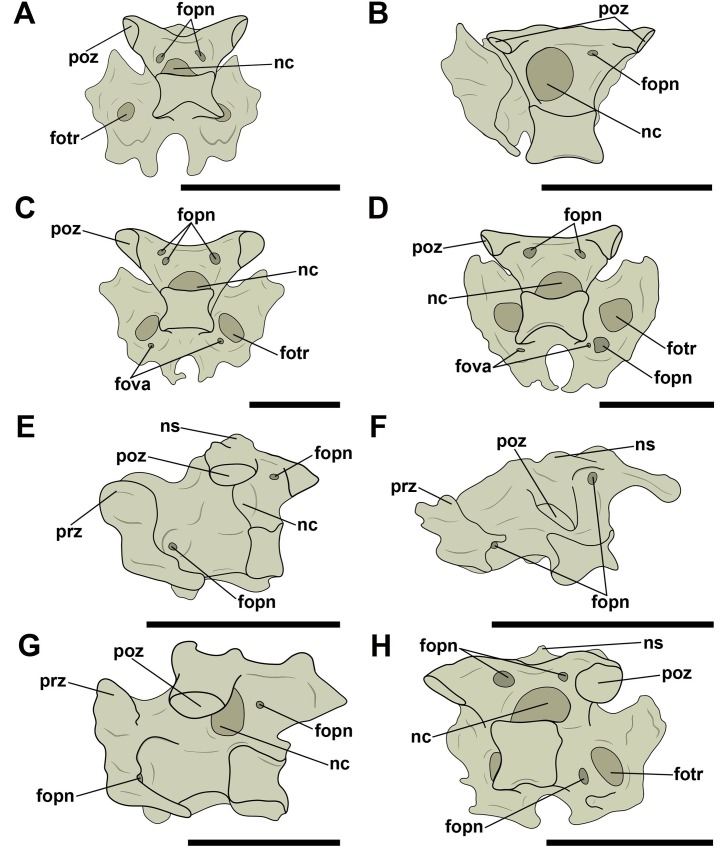
Pneumatic foramina located adjacent to the neural canal of mid-cervical vertebrae in birds. A, *Tinamus solitarius* (AZ 136) in caudal view; B, *Penelope superciliaris* (MNA 2058) in laterocaudal view; C, *Diomedea chlororhynchus* (MNA 1793) in caudal view; D, *Sula leucogaster* (MNA 7665) in caudal view; E, *Tyto furcata* (AZ 1543) in laterocaudal view; F, *Milvago chimachima* (MNA 5086) in laterocaudal view; G, *Ara chloropterus* (AZ 762) in laterocaudal view; H, *Ara macao* (MNA 001) in laterocaudal view. Abbreviations: fopn, pneumatic foramen; fotr, *foramen transversarium*; fova, vascular foramen; nc, neural canal; ns, neural spine; poz, postzygapophysis; prz, prezygapophysis. Scale: 10 mm.

On the mid-cervical vertebrae of many species of Neornithes, we noticed a presence of a pair of small pneumatic foramina dorsal to the neural canal in caudal view. Both tend to show a circular or oval outline (Fig 10). The amount of pneumatic foramina found in this position varied only in *Fregata magnificens* (MNA 1977), with an additional foramen on each side, and in *Diomedea chlororhynchus* (MNA 1793), with an additional foramen only on the left side, resulting in an asymmetrical arrangement in this case (Fig 10C). Three species belonging to Ardeidae (*Ardea alba*, *Egretta caerulea*, and *Egretta thula*) did not present pneumatic foramina dorsally adjacent to the neural canal, suggesting a probable character loss. The absence of the foramina in this position was also observed in other species, such as *Rhea americana* (MNA 766), *Pipile jacutinga* (AZ 767 and MNA 7664), *Procellaria aequinoctiallis* (MNA 8353), and *Catharthes aura* (AZ 578 and AZ 580).

#### Posterior cervical vertebrae

**Fig 11:**
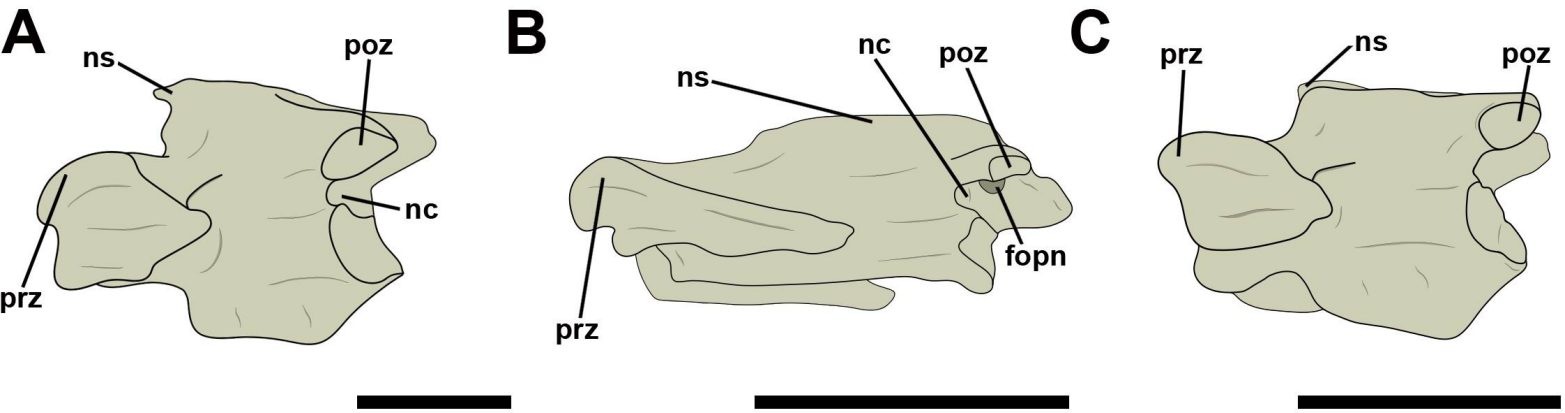
Posterior cervical vertebrae in ardeid birds in left lateral view. A, *Ardea alba* (MN 51321); B, *Egretta caerulea* (AZ 1073); C, *Egretta thula* (MNA 6993). Abbreviations: fopn, pneumatic foramen; nc, neural canal; ns, neural spine; poz, postzygapophysis; prz, prezygapophysis. Scale: 10 mm.

An increase in the number of pneumatic foramina on the posterior cervical vertebrae in relation to those observed in mid-cervicals is frequent. Most of the time, the increase occurred both on the lateral faces of centrum and neural arch. However, no pneumatic foramina are seen in the posterior-most cervicals or the anterior thoracic vertebrae of *Penelope superciliaris* (MNA 751 and MNA 2058) and in the analyzed Pelecaniformes, represented by species of Ardeidae (*Ardea alba*, *Egretta caerulea*, and *Egretta thula*). An exception are two pneumatic foramina that border the dorsal margin of the neural canal of the posterior cervical vertebrae of *Egretta caerulea* when seen in caudal aspect (Fig 11).

**Fig 12:**
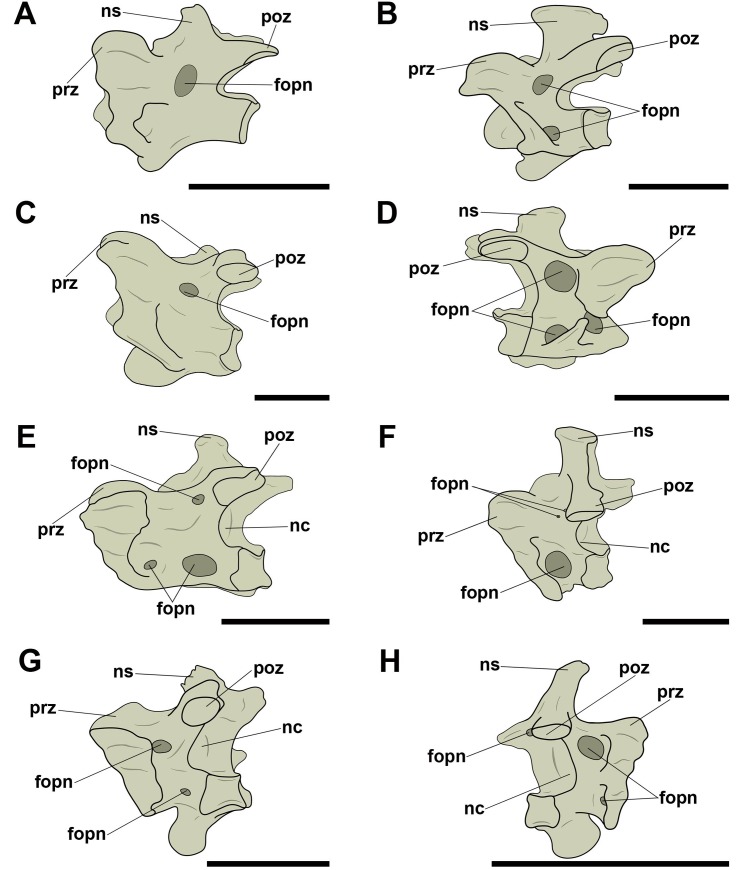
Pneumatic foramina located on the lateral surface of the neural arc and the centra of posterior cervical vertebrae in birds. A, *Tinamus solitarius* (AZ 139) in left lateral view; B, *Cairina moschata* (AZ 763) in caudolateral view; C, *Diomedea chlororhynchus* (MNA 1793) in left lateral view; D, *Procellaria aequinoctiallis* (MNA 8553) in caudolateral view; E, *Sula leucogaster* (MNA 7665) in caudolateral view; F, *Cathartes aura* (AZ 578) in caudolateral view; G, *Caracara plancus* (MNA 4902) in caudolateral view; H, *Falco sparverius* (AZ 1548) in caudolateral view. Abbreviations: fopn, pneumatic foramen; nc, neural canal; ns, neural spine; poz, postzygapophysis; prz, prezygapophysis. Scale: 10 mm.

Lateral pneumatic foramina on the centrum of posterior cervical vertebrae were present in 72% of the analyzed species. The craniolateral foramen was observed only in posterior cervical vertebrae. The occurrence of both foramina simultaneously in the vertebral centra varied: we observed species with a foramen only in craniolateral position, such as *Athene cunicularia* (AZ 1384), *Falco sparverius* (AZ 1547, AZ 1185, AZ 1548, AZ 1096, AZ 1092), and *Ara macao* (MNA 001), species which had only the lateral foramen, like *Cairina moschata* (AZ 763), *Pipile jacutinga* (AZ 767 and MNA 7664), *Fregata magnificens* (MNA 1977), *Catharthes aura* (AZ 578 and AZ 580), *Milvago chimachima* (MNA 4305 and MNA 5086), and *Caracara plancus* (MNA 4902 and MNA 4589), species which presented foramina in both positions, such as *Sula leucogaster* (MNA 766), *Procellaria aequinoctiallis* (MNA 8353), *Pterodroma* sp. (AZ 1196), *Asio clamator* (AZ 1190), and *Ara chloropterus* (AZ 762), and species with no foramina on the vertebral centrum, like the Paleognathae *Rhea americana* (MNA 776) and *Tinamus solitarius* (AZ 136, AZ 138, and AZ 139) (Fig 12).

Lateral pneumatic foramina on the neural arch were not observed in mid-cervical vertebrae, but were relatively common in posterior cervical vertebrae, except for species of Galliformes, Pelecaniformes, Strigiformes, and Psittaciformes (Fig 12). They were present near the base of the postzygapophyses and were observed only between the eighth cervical and the second thoracic vertebrae, which do not present well-developed lateral transverse processes. We observed a variation in size between different taxa; in *Catharthes aura*, for example, the foramina showed a reduced size in relation to the pneumatic foramina on the craniolateral surface of centrum, while they were considerably bigger in *Procellaria aequinoctiallis* (Fig 12F and 12D, respectively).

**Fig 13:**
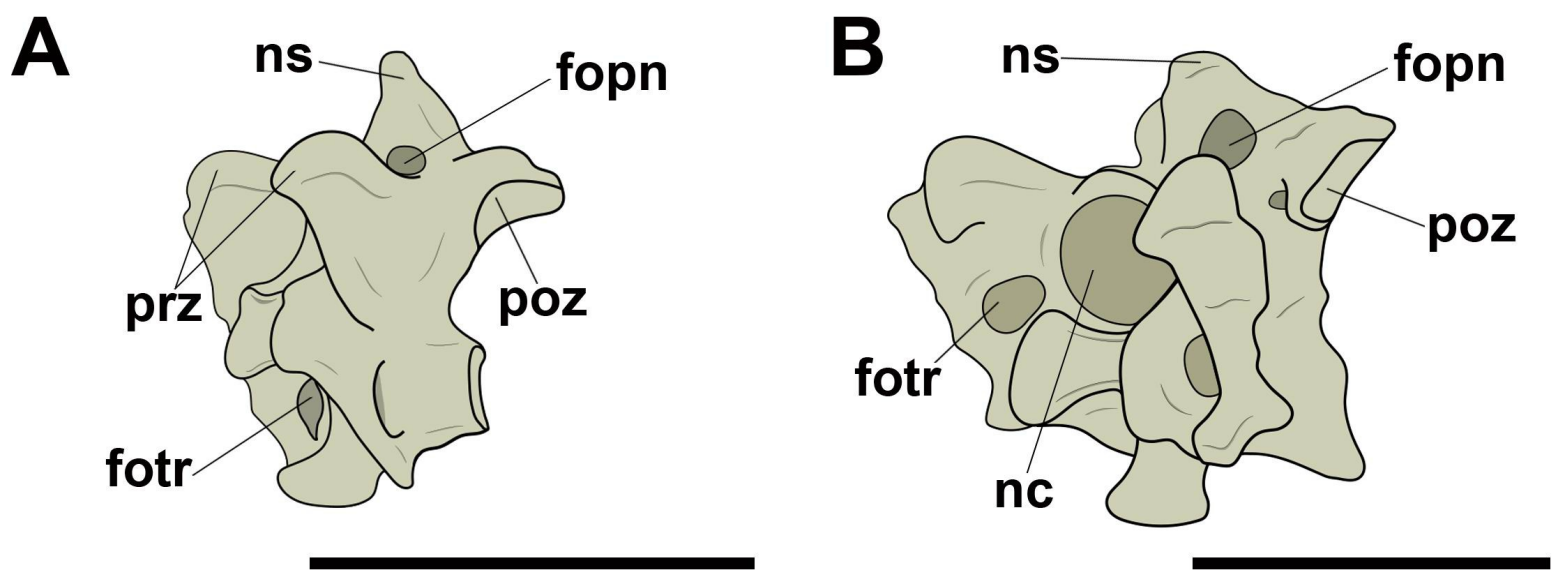
Pneumatic foramina located next to the base of the neural spine on the posterior cervical vertebrae in birds. A, *Milvago chimachima* (MNA 5086) in left lateral view; B, *Caracara plancus* (AZ 1297) in craniolateral view. Abbreviations: fopn, pneumatic foramen; fotr, *foramen transversarium*; nc, neural canal; ns, neural spine; poz, postzygapophysis; prz, prezygapophysis. Scale: 10 mm.

In Falconiformes, we observed pneumatic foramina with oval outlines located at the base of the neural spine on posterior cervical vertebrae (Fig 13).

#### Notarium

**Fig 14:**
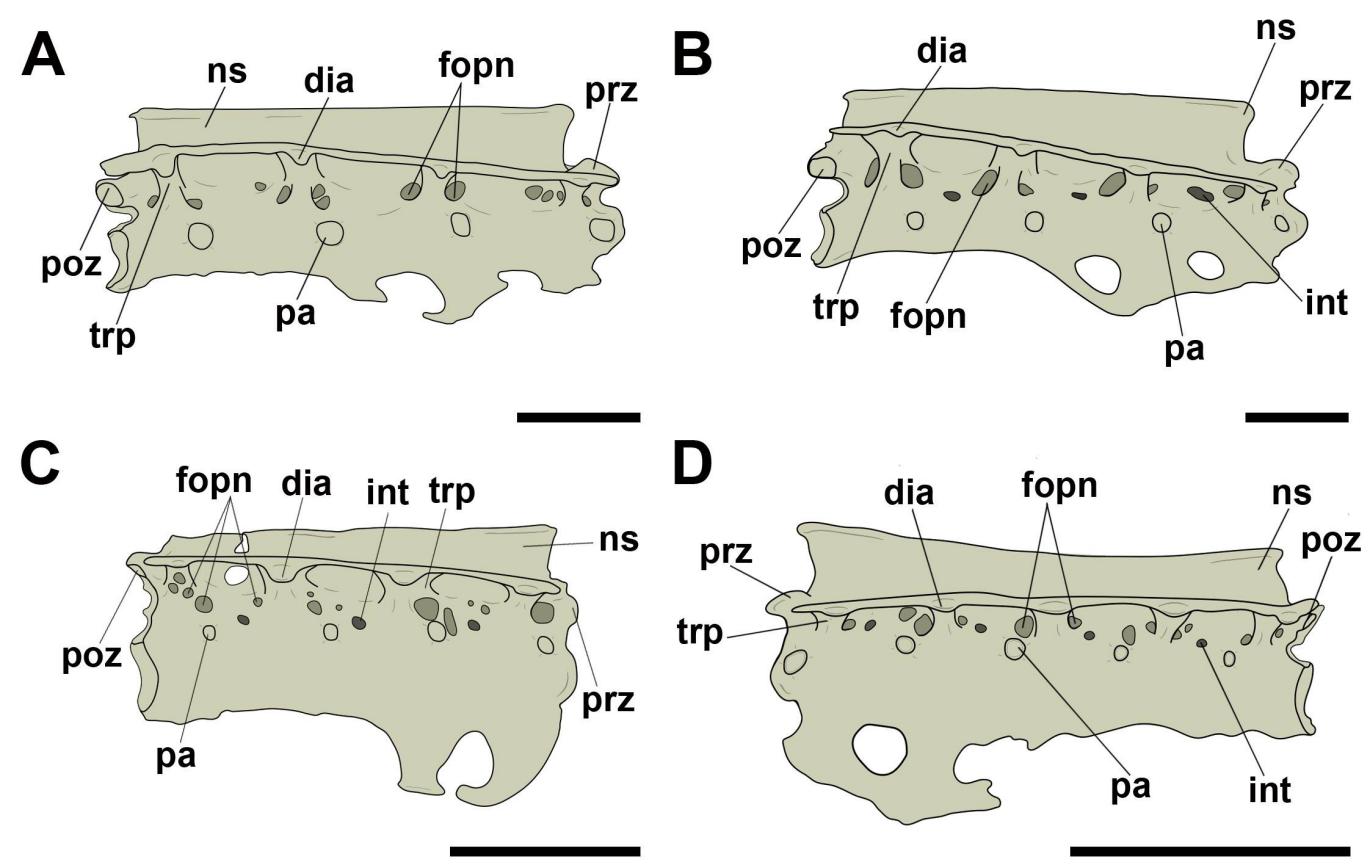
Pneumatic foramina located near the base of the transverse processes of the notarium in birds. A, *Penelope superciliaris* (MNA 2058) in right lateral view; B, *Pipile jacutinga* (MNA 7664) in right lateral view; C, *Milvago chimachima* (MNA 4305) in right lateral view; D, *Falco sparverius* (AZ 1548) in left lateral view. Abbreviations: dia, diapophysis; fopn, pneumatic foramen; int, intervertebral space; ns, neural spine; pa, parapophysis; poz, postzygapophysis; prz, prezygapophysis; trp, transverse process. Scale: 10 mm.

In all species in which the notarium was present, we observed pneumatic foramina positioned both cranioventrally and caudoventrally on the bases of the transverse processes (Fig 14). Such foramina did not follow a common pattern of number or shape. No analyzed species presented lateral pneumatic foramina on the vertebral centra.

#### Free thoracic vertebrae

**Fig 15:**
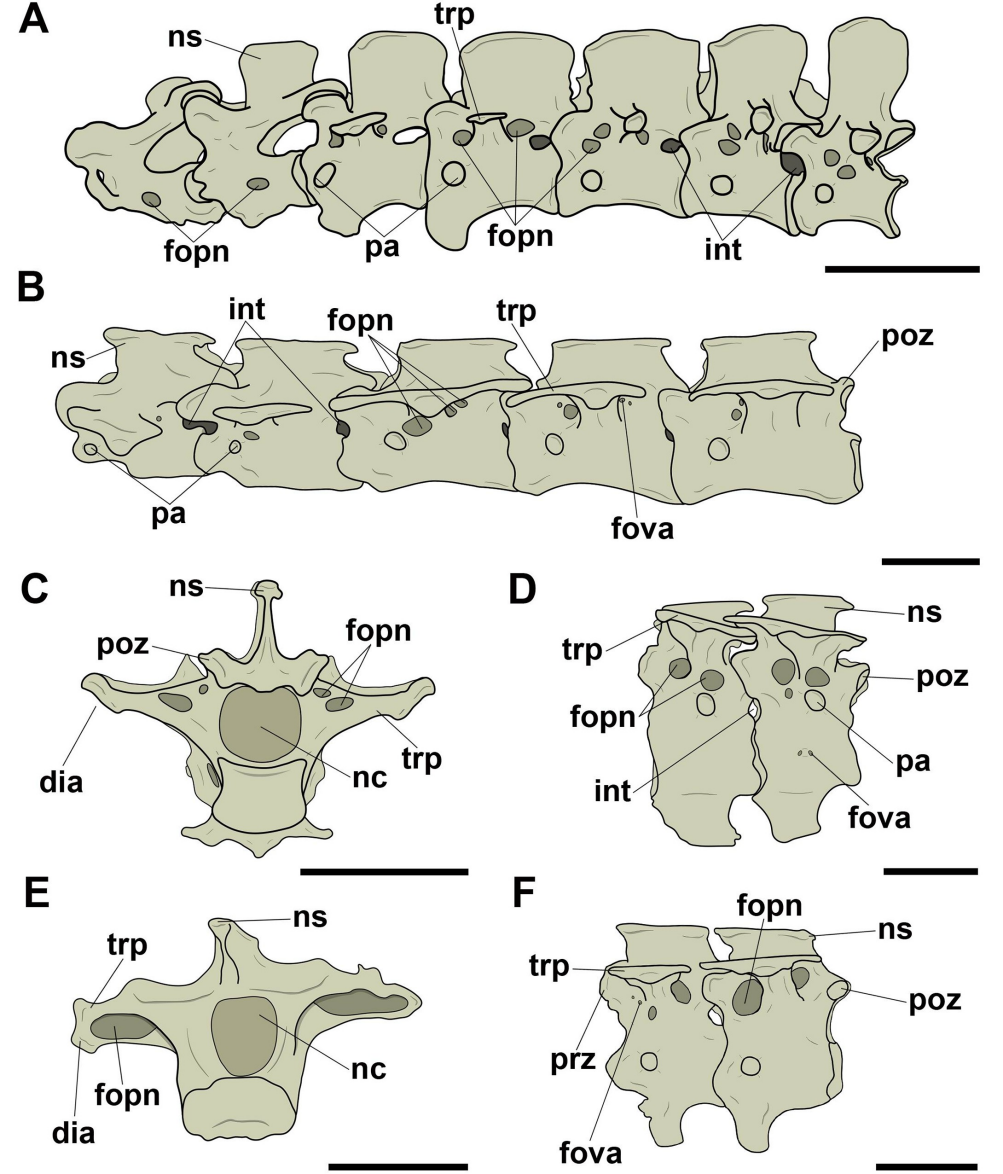
Pneumatic foramina located near the base of the transverse processes of the free thoracic vertebrae in birds. A, *Pipile jacutinga* (AZ 767) in left lateral view; B, *Ardea alba* (MNA 51321) in left lateral view; C, *Procellaria aequinoctiallis* (MNA 8553) in caudal view; D, *Cathartes aura* (AZ 578) in left lateral view; E, *Fregata magnificens* (MNA 1977) in cranial view; F, *Ara chloropterus* (AZ 762) in left lateral view. Abbreviations: dia, diapophysis; fopn, pneumatic foramen; fova, vascular foramen; int, intervertebral space; nc, neural canal; ns, neural spine; pa, parapophysis; poz, postzygapophysis; prz, prezygapophysis; trp, transverse process. Scale: 10mm.

We observed a varied number of pneumatic foramina at the bases of the transverse processes of the free thoracic vertebrae in all analyzed species. Certain clades had comparatively bigger foramina than others, like Procellariiformes, Suliformes, Accipitriformes, and Psittaciformes (Fig 15); in the case of *Fregata magnificens*, this structure extended to the end of the diapophysis (Fig 15E). Regarding their position, in all species there was at least one foramen cranially and one caudally in relation to the bases of the transverse processes.

**Fig 16:**
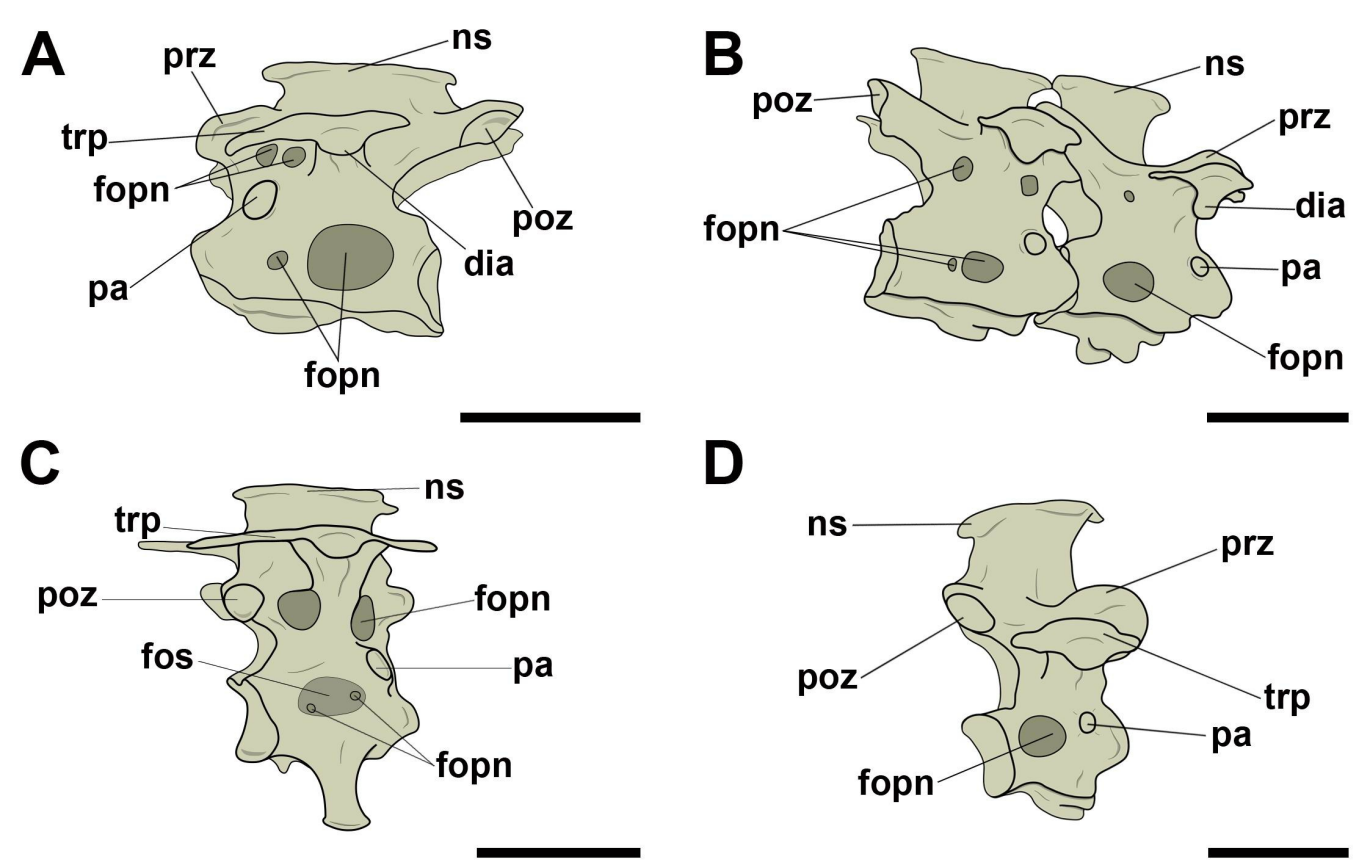
Pneumatic foramina located on the lateral surface of the centra of thoracic vertebrae in birds. A, *Sula leucogaster* (MNA 7665) in left lateral view; B, *Diomedea chlororhynchus* (MNA 1793) in right lateral view; C, *Procellaria aequinoctiallis* (MNA 8553) in right lateral view; D, *Ara ararauna* (AZUSP 040) in right lateral view. Abbreviations: dia, diapophysis; fopn, pneumatic foramen; fos, fossa; ns, neural spine; pa, parapophysis; poz, postzygapophysis; prz, pre-zygapophysis; trp, transverse process. Scale: 10 mm.

Lateral pneumatic foramina in the mid-length portion of the vertebral centra were observed only in Neoaves, being common in Procellariiformes (MNA 8553, MNA 1793), and are also present in Suliformes (*Sula leucogaster*, MNA 7665) and Psittaciformes (*Ara ararauna*, AZUSP 040) (Fig 16). These foramina are oval in outline, as are the ones on the centra of cervical vertebrae, although the thoracic ones are larger. Associated with them, we observed small pneumatic foramina in the analyzed Suliformes and Procellariiformes (see Fig 16A, 16B and 16C).

**Fig 17:**
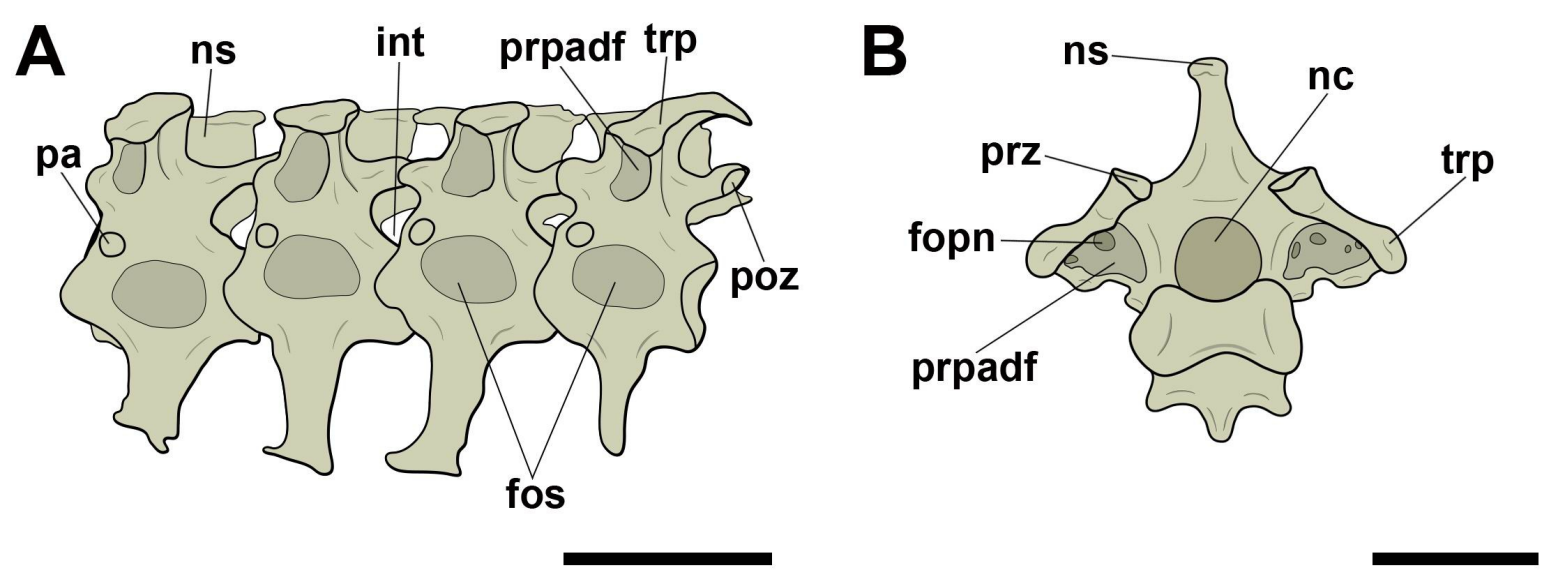
Prezygapophyseal parapodiapophyseal fossae and other pneumatic fossae observed on the surface of the free thoracic vertebrae in birds. A, *Pterodroma* sp. (AZ 1196) in left lateral view; B, *Ara ararauna* (AZUSP 040) in cranial view. Abbreviations: fopn, pneumatic foramen; fos, fossa; int, intervertebral space; nc, neural canal; ns, neural spine; poz, postzygapophysis; prpadf, prezygapophyseal parapodiapophyseal fossae; prz, prezygapophysis; trp, transverse process. Scale: 10 mm.

Some of the analyzed species also presented pneumatic fossae on the lateral faces of vertebral centra and/or prezygapophyseal parapodiapophyseal fossae (Fig 17), which vary in depth. In the specimen AZ 1196 (*Pterodroma* sp.) both are deep and without any association with pneumatic foramina; in MNA 009 (*Ara ararauna*), which only presents the prezygapophyseal parapodiapophyseal fossae, they are shallower and associated with pneumatic foramina on their interior; in MNA 8353 (*Procellaria aequinoctiallis*) we observed pneumatic fossae on the lateral faces of the centra, which also are pierced by small oval pneumatic foramina on their bottom (Fig 16C).

## Discussion

Analysis of pneumatic foramina in the vertebrae of Pterosauria and extant Neornithes demonstrates that pneumatization is unequivocally present along the cervical and dorsal/thoracic series of both clades, presenting the common pattern recognized for other Ornithodira, such as non-avian Neotheropoda [8, 24].

Variations in the pneumatic structures present on the cortical surface of the mid-cervical vertebrae were observed between Pterosauria and Neornithes. The pneumatic foramina observed on the lateral faces of the centra of mid-cervical vertebrae of all pterosaurs analyzed here are considered a synapomorphy for the Ornithocheiroidea [23], although this character was lost secondarily in the Azhdarchidae [23]. Furthermore, foramina analogous to those have been observed in non-ornithocheiroids, like *Dimorphodon macronyx* (NHMUK R1034) [25], suggesting an evolutionary convergence [26]. The variations in the number of the lateral pneumatic foramina observed here are consistent with that reported in the literature [27–29]. Foramina in craniolateral position on the centra of mid-cervical vertebrae were observed in all analyzed clades of Neornithes and are also observed on the cervical vertebrae of non-avian Averostra, as in *Baryonyx* (NHMUK R9951) and *Majungasaurus creatissimus* (UA 8678) [4, 24, 30]. They are seldom present in non-Averostra Neotheropoda, as in *Lilisternus* [31], although pneumatic foramina may be present more posteriorly on the cervical centrum, differing from the pattern seen in birds. In birds, the absence of the craniolateral foramen in some species could be explained as secondary losses, considering the topology of the phylogeny proposed by Yuri et al. [14]. When comparing lateral pneumatic foramina in pterosaurs and Neotheropoda, we observed that they differ in position and shape, being arranged more cranially in the latter, and exhibiting a more circular morphology in Neotheropoda and a craniocaudally long outline in pterosaurs [4, 24, 30, 31].

Pneumatic foramina on the lateral surfaces of the neural arch of mid-cervical vertebrae were described only in non-pterodactyloid pterosaurs, such as *Raeticodactylus filisurensis* (BNM 14524) [25], *Austriadraco dallavechiai* (BSP 1994 I 51) [25, 32], and *Sericipterus wucaiwanensis* (IVPP V14725) [33]. Among theropods, we observed foramina in the same position only in the Paleognathae, being absent in non-avian Neotheropoda and in the Neognathae [4, 8, 24, 30, 31], indicating a convergence between Pterosauria and Paleognathae. However, cervical vertebrae of Sauropodomorpha and posterior cervical vertebrae of non-avian Theropoda commonly present postzygapohyseal centrodiapophyseal fossae, as seen in the cervical vertebrae of *Rapetosaurus krausei* and *Majungasaurus crenatissimus* (UA 8678) [4, 30, 34], which are placed in the same position as the afore-mentioned pneumatic foramina, suggesting that they may have been derived from these pneumatic fossae.

The pneumatic foramina adjacent to the neural canal of the mid-cervical vertebrae of pterosaurs are present but not restricted to the clade Dsungaripteroidea [25], being also identified in the holotype of *Sericipterus wucaiwanensis* (IVPP V14725)[33]. The presence of three foramina adjacent to the neural canal (two laterally and one dorsally) observed in the analyzed Tapejaridae has also been previously described for Azhdarchidae [35, 36] and Pteranodontidae [19]. At least one adjacent foramen, either dorsal or lateral to the neural canal, was found in cranial and caudal views in the mid-cervical vertebrae of all observed pterosaurs, different from the pattern observed in birds, where they are only found in caudal view. Their number, size and position in relation to the neural canal also varied, with the foramina in birds located more dorsally, organized between the postzygapophyses, presented in pairs, and with very reduced size when compared to those present in pterosaurs. Pneumatic foramina adjacent to the neural canal as observed in birds followed the same patterns as in non-avian Neotheropoda in which these foramina were present, as in *Majungasaurus crenatissimus* (UA 8678) [4, 30].

In the posterior cervical vertebrae, we find a tendency to size reduction and loss of foramina located on the lateral sides of the centra of several pterosaur clades [19, 28, 37, 38], such as in the Azhdarchidae and the Ctenochasmatidae. In *Azhdarcho lancicollis*, a vestigial lateral pneumatic foramen was recognized on the specimen ZIN PH 137/44, an eighth cervical vertebra, but was absent on specimens ZIN PH 131/44, ZIN PH 144/44, ZIN PH 147/44, ZIN PH 138/44, interpreted, respectively, as third, fourth, sixth and seventh cervical vertebrae [35]. In contrast, there is no reduction of the craniolateral pneumatic foramina in Neotheropoda, including birds [4, 24, 30]. Additionally, a pneumatic foramen is present on the medial portion of the centra of the posterior cervical vertebrae of Neognathae, which resembles that seen in the respective vertebrae of pterosaurs. In birds, these foramina exhibited an oval outline, similar to the craniolateral foramina, although the size is considerably bigger.

Pneumatic foramina on the lateral surfaces of the neural arch of the posterior cervical vertebrae are reduced in size or absent in Pterosauria, but were frequently found in the Paleognathae, Anseriformes, Suliformes, Procellariiformes, and Falconiformes, in a position equivalent to that of the postzygapophyseal centrodiapophyseal fossae of non-avian Neotheropoda and Sauropodomorpha [22, 24, 30, 39].

Some of the species of pterosaurs and birds we analyzed here presented a notarium. There are records in the literature of the presence of pneumatic foramina on the bases of transverse processes in the notarium of pterosaurs, as in a specimen tentatively referred to *Santanadactylus brasilensis* and in *Anhanguera* sp. [28, 40, 41]. The position of these foramina resembles that of birds, in which they are arranged cranially and caudally to the bases of the transverse processes of the notarial vertebrae. However, the lack of pneumatic foramina in this structure in some large pterosaurs, as we observed in *Tropeognathus* cf. *mesembrinus* (MN 6594-V), was also described in the literature [19, 35], revealing the presence of pneumatic hiatuses along the cervical column, as anteriorly described for Neognathae and Sauropodomorpha [42–44]. The absence of foramina may be related to the fact that the function of the notarium is to confer stability and rigidity for this region of the column, which is susceptible to higher stresses due to the effort required by flight [45]. However, the absence of pneumatic foramina in the notarium was not observed in birds with large wingspan, so we cannot confirm here whether this could be linked to a biomechanical advantage. The pneumatic hiatus observed in the notarium of some pterosaurs resembles what Hogg [46] described for some birds, in which the gap occurs in thoracic vertebrae II and III. The presence of this pneumatic hiatus was attributed to the distance between thoracic vertebrae II and III and the cervical air sac, which would probably be responsible for their pneumatization [43, 46]. We speculate that the same possibly happened to the notarium of some pterosaurs.

Differing from what was observed in the notarium, the free dorsal vertebrae of pterosaurs presented pneumatic foramina on the margins of the bases of the transverse processes, which were already reported in the Pteranodontoidea [9, 11, 19, 29] and the Azhdarchoidea [35, 47, 48], being similar both in form and number to the taxa analyzed here. Pneumatic foramina in similar position were observed in all analyzed Neornithes, and can be explained from a biomechanical point of view, because these are areas where their presence does not compromise the physical integrity of the vertebra [3]. According to the literature, pneumatic foramina are also present on the bases of the transverse processes of dorsal vertebrae attributed to non-avian Neotheropoda, such as in *Majungasaurus crenatissimus* (UA 8678) [4, 5, 30]. In Sauropodomorpha, the prezygapophyseal centrodiapophyseal fossae, centrodiapophyseal fossae, and postzygapophyseal centrodiapophyseal fossae are arranged in equivalent places, as observed in *Plateosaurus* and *Camarasaurus supremus* [8, 24]. The widespread presence of pneumatic foramina and/or fossae at the bases of the transverse processes in ornithodirans known to have pneumatization suggests competing hypotheses: that vertebral pneumatization in this site may have a homologous origin in these groups, that this is a biomechanically favored position, or both.

Despite the absence of pneumatic foramina on the lateral faces of the centra of the free dorsal vertebrae in the pterosaurs analyzed here, they have been previously observed in non-pterodactyloids, such as in the third dorsal vertebra of the holotype of *Raeticodactylus filisurensis* [25], and in the anterior-most five preserved dorsal vertebrae of the specimen GSM 1546, identified as *Dimorphodon macronyx* [25]. These pneumatic foramina present a craniocaudally long morphology and mid-length position in the vertebral centrum, similar to those seen in pterosaur mid-cervical vertebrae. In contrast, the lateral pneumatic foramina of the thoracic vertebrae of Neoaves differ from those of Pterosauria because they have an oval outline and larger diameters. In Procellariformes and Suliformes, they were associated with other small pneumatic foramina, which did not follow a common pattern in size and position. Non-avian Neotheropoda, such as *Torvosaurus* (BYU 2008) [24], also have equivalent pneumatic foramina with a circular shape similar to that observed in birds. The absence of any pneumatic characteristic on the centra of the free dorsal vertebrae of Tinamiformes, Rheiformes, Anseriformes, and Galliformes thus suggests secondary losses.

Despite the few records of pneumatic fossae in this work, in the literature, the presence of prezygapohyseal parapodiapophyseal fossae and of postzygapophyseal centrodiapophyseal fossae in pterosaurs has previously been observed on the dorsal vertebrae of Rhamphorhynchidae. These fossae had no connection with the internal cavities in the holotype of the rhamphorhynchid *Sericipterus wucaiwanensis* (IVPP V14725) [33]. In *Rhamphorhynchus muensteri* (MGUH 1891.783), these fossae are associated with pneumatic foramina [37] similar to those observed in the prezygapophyseal parapodiapophyseal fossae of the free thoracic vertebrae belonging to the procellariiform *Pterodroma* sp. (AZ 1196). The pneumatic fossae of birds and pterosaurs resemble, in position and appearance, the prezygapophyseal parapodiapophyseal fossae and postzygapophyseal centrodiapophyseal fossae present in the dorsal vertebrae of sauropodomorphs, exhibiting a concavity delimited by vertebral laminae [22], thus suggesting deep homologies.

Pneumatic foramina present in unusual locations in certain clades, such as near the neural spine in the cervical vertebrae VIII in thalassodromine pterosaurs and in the posterior cervical vertebrae of Falconiformes, and unusually absent, such as on the lateral faces of the posterior cervical vertebrae in the Ardeidae and on the lateral faces of the mid-cervical vertebrae in the Ctenochasmatidae and the Azhdarchidae, can be interpreted as independent gains or losses in these clades [23, 26].

Variations on the size of pneumatic foramina are observed along the vertebral column of the same individual, with the largest foramina generally closer to the air sac or lung that invades the bone [4, 5, 30]. We observed size differences which therefore may indicate different origins of the air diverticula in the neck of birds and pterosaurs. Birds show an increase in the size and/or quantity of pneumatic foramina from the mid-cervical to the posterior cervical vertebrae, as also seen in the theropods *Majungasaurus*, *Spinostropheus*, *Allosaurus*, *Monolophosaurus*, and *Sinraptor* [4, 30]. The opposite is observed in pterosaurs, a difference that indicates that their cervical air sac had a robust cranial portion, unlike theropods.

Procellariiformes, Suliformes, and Falconiformes have more numerous and larger pneumatic foramina than the other analyzed birds. These birds perform static soaring [15–18], an ecological habit previously recognized as associated with the development of increased pneumatization for its energy saving in locomotion and foraging [49]. Among all analyzed species, only *Phalacrocorax brasilianus* (Phalacrocoracidae, Suliformes) did not show any evidence of pneumatization in the axial postcranial skeleton, which may be related to its aquatic foraging habit, since less dense bones interfere with submersion, as is reported for penguins, loons and diving ducks [3], and/or to the presence of their extremely compact vertebral centra.

According to O’Connor [4], in birds with pneumatic vertebrae, the same foramen may play the role of pneumatization and vascularization. In the cervical vertebrae, the vertebral vascular foramina accommodate the *arteria vertebralis*. They can be present as a single foramen, as in *Diomedea chlororhynchus* (MNA 1793) and *Sula leucogaster* (MNA 7665), or subdivided into several smaller ones, without a standard shape, as in *Rhea americana* (MNA 766) and *Ara ararauna* (AZUSP 040) (Fig 8A and 8B). They can be located on the craniolateral faces of the centrum, similarly to the pneumatic foramina common to most birds observed here. In non-avian Neotheropoda, the foramina on the craniolateral faces of the centrum in non-pneumatic cervical vertebrae have vascular function [4]. In the thoracic vertebrae, the vascular foramina house the *arteria intercostalis dorsalis* [50]. We observed vascular foramina smaller than the pneumatic ones on the ventral bases of the transverse processes of *Ardea alba* (MNA 51321) and *Ara chloropterus* (AZ 762) (Fig 15B and 15F). However, although generally smaller, the sizes of vascular and pneumatic foramina may be similar. Therefore, although there are no external morphological differences between vascular and pneumatic foramina other than the presence of a connection between the foramen and intertrabecular cavities [4], the positions of pneumatic foramina arranged in cervical and dorsal/thoracic vertebrae in pterosaurs and birds seem to be consistent in different clades, which may allow their identification in future descriptive analyses.

## Conclusions

The differences between positions, sizes, shapes and quantity of the pneumatic features present on the cortex of the cervical and dorsal/thoracic vertebrae of pterosaurs and birds suggests probable convergences in the development of the pneumatic foramina presented in the vertebral column of ornithodirans, either due to biomechanical influences or by the ecological habits performed.

In both pterosaurs and birds, we observed presence and absence of pneumatic foramina in unusual places in certain groups, which were interpreted as independent acquisitions and losses in these groups, corroborating previous observations [27]. These variations, as well as differences in number and size of the foramina associated with a particular region of the vertebral column, are related to the different body sizes and/or life habits of each species [3].

The variation in the size and/or quantity of pneumatic foramina along presacral vertebrae, which decreased from the mid-cervical to the posterior cervical vertebrae of Pterosauria, and exhibited the opposite trend in Neotheropoda, is interpreted here as a possible variation of the cervical air sac between these two clades, which is probably more robust cranially in pterosaurs. In addition, the pneumatic hiatus associated with to the apneumatic notarium observed in some pterosaurs is probably due to the distance of this structure from the cervical air sac, which is probably the one responsible for the formation of pneumatic diverticula in this area of the vertebral column.

Although it was not possible to differenciate externally pneumatic and vascular foramina, we observed that the position of vascular foramina is probably the best feature to differenciate both, since their location is less variable.

## Abbreviations

AMNH: American Museum Natural History, New York, USA
AZ/AZUSP: Anatomical Collection of the Ornithology Division, Department of Zoology, University of São Paulo, Brazil
GSM: Museum of the Geological Survey, Keyworth, United Kingdom
IVPP: Institute of Vertebrate Paleontology and Paleoanthropology, Beijing, China
MN: Paleovertebrate Sector, Department of Geology and Paleontology, National Museum/Federal University of Rio de Janeiro, Brazil
MNA: Anatomical Collection of the Ornithology Sector, Department of Vertebrates, National Museum/Federal University of Rio de Janeiro, Brazil
NHMUK: Department of Palaeontology, The Natural History Museum, London, United Kingdom
UA: University of Antananarivo, Madagascar
ZIN PH: Paleoherpetological Collection, Zoological Institute of the Russian Academy of Sciences, Saint Petersburg, Russia
